# Adsorption of Aqueous Nickel Ion by Biomass Carboxymethyl Cellulose-Doped Boron Nitride Composites and Its Subsequent Energy Storage

**DOI:** 10.3390/polym17050567

**Published:** 2025-02-20

**Authors:** Xinran Li, Boyun Wang, Wanqi Zhang, Xiaotao Zhang, Ximing Wang

**Affiliations:** 1 College of Science, Inner Mongolia Agricultural University, Hohhot 010018, China; 2 College of Material Science and Art Design, Inner Mongolia Agricultural University, Hohhot 010018, China; 3 Inner Mongolia Key Laboratory of Sandy Shrubs Fibrosis and Energy Development and Utilization, Hohhot 010018, China; 4 National Forestry Grassland Engineering Technology Research Center for Efficient Development and Utilization of Sandy Shrub, Hohhot 010018, China

**Keywords:** Ni^2+^, carboxymethyl cellulose, biomass gel, resource utilization, double-layer capacitor

## Abstract

As a typical heavy metal pollutant discharged from industrial activities, nickel ions are highly bioaccumulative and carcinogenic, and low concentrations (>0.5 mg/L) can disrupt the balance of aquatic ecosystems and pose a threat to human health. In this study, a bifunctional adsorbent based on a carboxymethyl cellulose/boron nitride hydrogel was prepared for the treatment of nickel-containing wastewater with a high adsorption capacity of Ni^2+^ (800 mg/L, 344 mg/g), and after adsorption, the waste gel was converted into nickel-doped porous carbon material through carbonization and used as a bilayer capacitor electrode to achieve a specific capacitance of 40.6 F/g at a current density of 1 A/g. The capacity retention rate was >98% after 150 cycles. This strategy simultaneously solves the problems of nickel-containing wastewater purification (the adsorption method is applicable to medium- and high-concentration heavy-metal wastewater) and environmental pollution caused by waste adsorbents, and provides a new paradigm of the “adsorption-resourcing” closed-loop treatment of heavy-metal pollutants.

## 1. Introduction

Rapid population growth and industrialization have resulted in excessive levels of hazardous pollutants in aquatic environments, causing irreversible negative impacts on the ecosystem [1]. Among the many harmful pollutants, heavy metals have become a focus of public attention because they cannot be eliminated through biodegradation. Lead enters the human body through drinking water or aquatic organisms, preferentially accumulates in bones and kidneys, interferes with hemoglobin synthesis and induces anemia. Cadmium damages the human kidney and skeletal system, and long-term intake of cadmium-containing aquatic products may induce lung cancer and prostate cancer. Hexavalent chromium has strong oxidizing and carcinogenic properties, which can damage DNA structure and induce lung cancer [2]. Ni^2+^, as a harmful heavy metal, is widely found in the mining industry [3], metal smelting industry and in battery manufacturing [4]. The discharge of Ni^2+^-containing pollutants causes direct or indirect pollution of the atmosphere, water resources and soil. Ni^2+^ in wastewater mainly exists in the form of divalent cations, and the prolonged exposure of animals to Ni^2+^ poses a serious health hazard. Long-term intake of nickel-containing water can lead to damage to organs such as the heart, brain and liver [5]. A variety of solutions have been developed for removing Ni^2+^ from wastewater systems, including through adsorption, membrane separation [6], chemical precipitation and ion exchange [7]. Among these methods, adsorption is widely considered to be an effective measure for removing pollutants from wastewater systems, and has advantages regarding the simplicity of operation, low cost and the ease of obtaining adsorbent materials [8].

China is a large agricultural country which produces a large amount of agricultural and forestry wastes every year, and cellulose is one of the most important components of those wastes. Carboxymethyl cellulose, as one of the important derivatives of cellulose, is widely used in industrial production. In addition, carboxymethyl cellulose-based hydrogels are widely used in heavy metal ion adsorption studies [9]. Compared with natural cellulose, carboxymethyl cellulose and its derivatives are grafted with a large number of hydroxyl, carboxyl and other reactive groups with excellent adsorption properties, which are conducive to increasing the cation exchange capacity of carboxymethyl cellulose-based hydrogels in heavy metal adsorption processes. A composite hydrogel made by Zeng Ruiqi [10] was prepared by introducing fish gelatine and bamboo shoot particles (BSPs) into the carboxymethyl cellulose (CMC) hydrogel in order to improve the strength of the hydrogel and its heavy metal ion removal capacity. The composite hydrogel was shown to be effective in removing Cd^2+^, Hg^2+^ and Pb^2+^ from aqueous solution and exhibited good recoverability. A quasi-secondary kinetic model described the physical adsorption of heavy metals by the composite hydrogel, and the Langmuir model showed that the maximum adsorption capacities were 147.7 mg/g for Cd^2+^, 88.62 mg/g for Hg^2+^ and 163.89 mg/g for Pb^2+^.

The lamellar structure of boron nitride consists of sp^2^-hybridized covalent bonds connecting a boron (B) atom and a nitrogen (N) atom, while the layers interact with each other through relatively weak van der Waals forces [11]. This structure gives boron nitride excellent chemical stability, a high specific surface area and a rich pore structure, which has made research on the performance of adsorbing heavy metal ions a hot topic [12]. Recent studies have further confirmed the potential application of boron nitride in the field of adsorbing heavy metal ions. Dong Peng [13] found that the maximum adsorption capacities of boron nitride flakes were 625.0 mg/g for Hg^2+^ and 210.97 mg/g for Pb^2+^, in accordance with the Langmuir model of adsorption for adsorption on a single molecular layer. The adsorption process was a quasi-secondary kinetic process of chemisorption.

Converting the biomass hydrogel materials produced after the adsorption of heavy metals into functional materials for secondary utilization can not only alleviate the hazards of heavy metals to human health but also solve the environmental pollution problem posed by waste adsorbents. The previously mentioned technical route is more practical and feasible [14]. In order to utilize the carboxymethyl cellulose-based hydrogel after the adsorption of heavy metals more effectively and convert it into energy storage materials, electrochemical technology is critical. Among most electrochemical energy storage devices and equipment, supercapacitors have attracted much attention because of their excellent energy storage properties. The most important part of supercapacitors is the electrode material, and porous carbon materials are widely used in this regard. Porous carbon materials have many advantages, such as having a high porosity structure, large specific surface area and excellent electrical conductivity [15]. Porous carbon materials need to meet the goals of cost control, zero pollution and simple synthesis steps when applied to energy storage devices. The preparation of carbon materials from biomass waste is advantageous because it is environmentally friendly, sustainable, and resource-based. In recent years, renewable biomass waste has been increasingly investigated as a carbon precursor, and Ni^2+^ is considered to have great potential in electrochemistry. Xin Zhao [16] modified carbon fiber materials with Ni^2+^ nanoparticles, which exhibited a high specific capacitance of 268 F/g and good cycling stability in 6 M KOH solution and at a current density of 0.2 A/g. Sun-I Kim [17] prepared flexible three-dimensional nickel (3D-Ni) electrodes; the Ni(OH)_2_/3D-Ni anode exhibited an excellent specific capacitance of about 3400 F/g at 10 A/g and maintained 80% of the initial capacitance at 200 A/g.

In addition to the above, proper doping of the heteroatom is also an effective way to optimize the structure of carbon-based materials. An increase in the doping of heteroatoms such as S, B, N and P changes the electronic structure of carbon materials and helps to improve the ionic conductivity and surface chemistry of carbon materials [18]. Porous carbon materials increase their own chemically active sites. As a result, heteroatom doping can increase the electronic conductivity and the induced pseudocapacitance [19]. In addition, diatom-doped carbon materials can increase surface chemical activity and improve electrochemical reaction mechanisms, and can also affect the catalytic properties and chemical reaction performance of carbon materials through synergistic effects [20]. Boron nitride materials have been used to improve the electrochemical performance of solid-state electrolytes in lithium-metal batteries [21], and boron nitride has attracted the attention of researchers due to its high theoretical energy density and excellent electrochemical properties [22]. Chen Xiufang [23] proposed a B/N-doped bamboo-derived carbon to realize a graded porous structure of bamboo-derived carbon materials to enhance the high electrochemical activity and further enhance electrical conductivity via co-doping with nitrogen and boron. The carbon material electrode exhibited significant energy density (37.8 Wh/kg in 1 mol/L KOH) compared with most related material electrodes.

In this study, boron and nitrogen co-doped biomass hydrogels were prepared based on carboxymethyl cellulose, using boron nitride materials as boron and nitrogen sources, which were used as adsorbents to adsorb heavy metal Ni^2+^ ion from water. The experiment mainly investigated the effects of Ni^2+^ ion concentration, water pH, adsorption time and adsorption temperature on the adsorption performance. In addition, the structure of hydrogel and its interaction mechanism with heavy metal ions were explored. The feasibility of the waste Ni^2+^ adsorbent as an energy storage material was evaluated by recycling it. The conversion of the waste adsorbent into electrode material after the adsorption of Ni^2+^ was realized at 600 °C under a nitrogen atmosphere. This work avoids the secondary hazards of the waste adsorbent and provides new technology for the treatment of water pollution.

## 2. Experimental Section

### 2.1. Materials

NaOH, urea, epichlorohydrin (ECH) and NaCl were purchased from Tianjin Kemao Chemical Reagent Company Limited, Tianjin, China. NiCl_2_-6H_2_O, boron nitride nanoscale (BNNS), KOH and carboxymethylcellulose (CMC) were purchased from Shanghai Macklin Biochemistry and Technology Company Limited, Shanghai, China. Deionized water was obtained from the laboratory.

### 2.2. Preparation of Carboxymethyl Cellulose Boron Nitride Hydrogels

BNNS was homogeneously dispersed in an aqueous solution of sodium hydroxide urea, and CMC was added to this solution in small amounts several times during stirring with the mass ratio of each substance as (CMC:BNNS:NaOH:Urea: H_2_O = 5:7:12:81). It was fully dissolved three times through the freezing and thawing method, and then cross-linking was undertaken with epichlorohydrin as a cross-linking agent (the volume ratio of the mixture was 1:10) at 55 °C for 6 h to complete the polymerization. Afterwards, the resulting gels were rinsed repeatedly with deionized water to remove unreacted chemicals and freeze-dried for 48 h to obtain CMC-BNNS hydrogels [24].

### 2.3. Adsorption Experiments

Ni^2+^ adsorption was carried out by taking 50 mg of CMC-BNNS hydrogel and placing it in 50 mL of Ni^2+^ solution in a thermostatic water bath oscillator to investigate the effects of Ni^2+^ solution concentration, adsorption time, adsorption temperature and adsorption water pH on the adsorption effect of the gel. After the completion of adsorption, the supernatant (0.45 µm) was removed with a filter membrane in a flame atomic absorption spectrometer (PinAcle 900F, PerkinElmer, Waltham, MA, USA) to assess the concentration of Ni^2+^ in the filtrate, and the effect of adsorption was evaluated by the following equation [25]:

$$q_{t}=(c_{0}-c_{t})V_{0}/M_{0}$$

where *q_t_* (mg/g) denotes the adsorption capacity at *t* (min), *c*_0_ (mg/L) is the initial Ni^2+^ concentration, *c_t_* (mg/L) is the Ni^2+^ concentration after adsorption, *V*_0_ (L) is the volume of the solution and *M*_0_ (G) is the mass of CMC-BNNS.

Pseudo-first-order (PFO) and pseudo-second-order (PSO) models are widely used as data-fitting models for the evaluation of adsorption processes [26].

PFO models:

$$q_{t}=q_{e}(1-e^{-k_{1}t})$$

PSO models:

$$q_{t}=\frac{k_{2}q_{e^{2}}t}{1+k_{2}q_{e}t}$$

where *q_e_* and *q_t_* are the amounts of heavy metal ions adsorbed (mg/g) at equilibrium and at time *t* (min), respectively; *k*_1_ (min^−1^) is the pseudo-first-order rate constant; and *k*_2_ [g·(mg/min)^−1^] is the rate constant of the pseudo-second-order adsorption kinetic equation.

### 2.4. Heat Treatment

The adsorbed saturated CMC-BNNS hydrogels were placed in KOH solution and vacuum impregnation was used to assist in the complete incorporation of KOH into the hydrogel until the hydrogel did not float. After freeze-drying, the hydrogel was activated in a tube furnace at a ramp rate of 5 °C per minute to 300 °C for two hours. The heating rate was continued at 5 °C per minute to 600 °C for 4 h. The concentrations of KOH when impregnated were 0.3 mol/L and 0.5 mol/L, respectively, and the materials were named C-BN-Ni-K3 and C-BN-Ni-K5; the unimpregnated material was C-BN-Ni, which was directly heated up to 600 °C and kept for 4 h. This material was named C-BN-Ni-K3-600.

### 2.5. Electrochemical Testing

The electrochemical performance was evaluated in the electrochemical laboratory using the Shanghai Chenhua electrochemical workstation and the three-electrode system. During the experiments, a platinum sheet was used as an auxiliary electrode, a mercury/mercury oxide electrode was used as the reference electrode and a potassium hydroxide solution of 6 molar concentration was chosen as the electrolyte. For the preparation of the working electrode, the material, polytetrafluoroethylene (PTFE) and acetylene black were mixed in an 8:1:1 mass ratio and stirred thoroughly with the help of ethanol to form a homogeneous paste. Subsequently, the paste was coated on a nickel foam substrate (1 cm × 1 cm in size) and compacted under a pressure of 20 MPa for 2 min. Cyclic voltammetry curves were obtained by cyclic voltammetry (CV) in the potential range of −1 V to 0 V. These curves demonstrate the reversibility of the charging/discharging process. The potential plateau, capacitance characteristics and current density of the materials were directly observed by constant current charge/discharge (GCD) testing. In addition, the charging/discharging behavior of the material at different current densities was evaluated and the relevant parameters were calculated using specific formulas [27].

$$C_{p}=I\;\Delta t/m\;\Delta V$$

where *I* is the constant current (A), *m* is the mass load (g), *t* is the discharge time (s) and *V* denotes the potential window (V). Electrochemical impedance spectroscopy (EIS), which reflects the conductivity of the material, the electron transfer resistance at the electrode–electrolyte interface and the mass transfer resistance of the liquid phase, was obtained in the frequency range of 1 Hz~100 k Hz and at a voltage of 5 mV.

## 3. Result and Discussion

### 3.1. Design and Characterization of Carboxymethyl Cellulose Hydrogels

**Figure 1:**
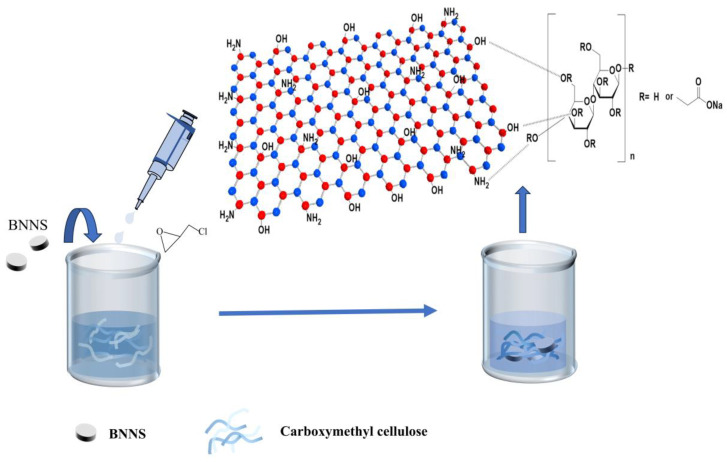
Preparation method of CMC-BNNS gel material.

The schematic diagram in Figure 1 shows the synthesis of BNNS hydrogels supported by a carboxymethylcellulose skeleton. The surface of CMC contains a large number of hydroxyl and carboxyl groups, and the BNNS has hydroxyl and amino groups at the edges and in its interior [28]. In this case, hydrogen bonding from the large number of surface hydroxyl/carboxy groups between the two compounds was achieved to allow the BNNS to easily adsorb onto the carboxymethylcellulose backbone. Carboxymethyl cellulose acts as a bridge connecting the BNNS [29]. Epichlorohydrin was used as a carboxymethyl cellulose cross-linking agent to obtain CMC-BNNS hydrogels. The epoxy group of epichlorohydrin underwent a ring-opening reaction with the hydroxyl group of CMC to form an ether bond. This process took place under alkaline conditions, where the base catalyzed the ring opening of the epoxy groups and facilitated the cross-linking reaction to obtain a CMC-BNNS hydrogel [30]. Epichlorohydrin reacted with the hydroxyl groups of the CMC, while BNNS was embedded in the CMC network by physical or chemical action to enhance the mechanical properties of the hydrogel. The aim was to obtain a highly porous hydrogel, such that the carboxymethyl cellulose and BNNS mixture retained a stable gelatinous dispersion until crosslinking. The hydrogel-like structure was then freeze-dried to obtain CMC-BNNS hydrogels.

**Figure 2:**
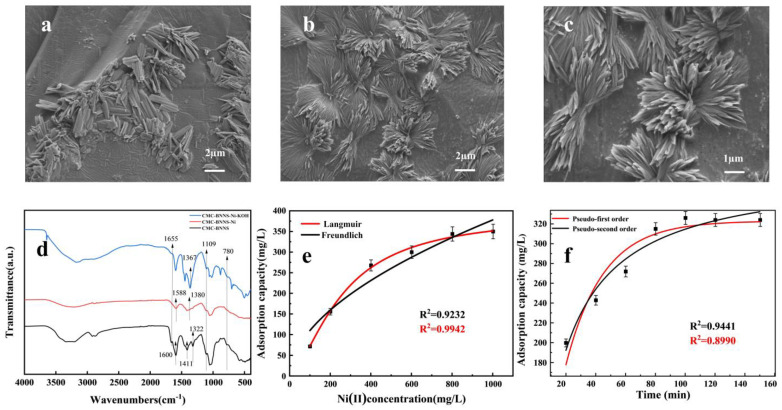
(**a**–**c**) CMC-BNNS SEM images. (**d**) FTIR spectra before and after Ni^2+^adsorption. (**e**) Adsorption isotherm nonlinear fitting curves of the Langmuir model and Freundlich model. (**f**) Adsorption kinetic nonlinear fitting curves of the pseudo-first-order model and pseudo-second-order model.

Figure 2a–c show the SEM images of the carboxymethylcellulose hydrogel, and the flower-like BNNS can be clearly seen attached to CMC [31]. As show in Fourier-transform infrared (FTIR) spectroscopy (Figure 2d), the results of CMC-BNNS show O-H telescopic vibration absorption peaks in the carboxymethyl cellulose structure at 3343 cm^−1^ and 3207 cm^−1^, C-H telescopic vibration absorption peaks in the carboxymethyl cellulose structure at 2922 cm^−1^ and 2875 cm^−1^, carboxyl C=O telescopic vibration absorption peaks in the carboxymethyl cellulose structure at 1665 cm^−1^ and carboxymethyl cellulose C=O telescopic vibration absorption peaks in the carboxymethyl cellulose structure at 1109 cm^−1^. In the C-O stretching vibration absorption peak in the structure of carboxymethyl cellulose [32], 1322 cm^−1^ is the stretching vibration absorption peak of N-B of boron nitride material and 780 cm^−1^ is the bending vibration absorption peak of N-B of boron nitride material [33]. After the adsorption of Ni^2+^ on the CMC-BBNS hydrogel, the carboxymethyl cellulose carboxylic acid C=O absorption peak disappeared at 1665 cm^−1^, which indicates that the carboxylic acid group and Ni^2+^ generate intermolecular forces. The asymmetric stretching vibration (ν_a_s) of the carboxylic acid group of the pristine CMC is located at 1600 cm^−1^ and the symmetric stretching vibration (ν_a_s) is located at 1411 cm^−1^, and after the adsorption of nickel, both peaks shifted to the lower-frequency direction (ν_a_s shifted to 1588 cm^−1^ and ν_s_ shifted to 1380 cm^−1^), suggesting that the electron cloud density of the carboxylic acid group decreases, ligand bonding with Ni^2+^ is formed and the intensity of the peaks weakens due to Ni^2+^ coordination, leading to the limitation of the vibrational freedom of the carboxylic acid group. Furthermore, the N-B bond and the C-H and O-H bond peak strengths in carboxymethyl cellulose exhibit a significant decreasing trend, which suggests that these groups are also involved in the adsorption reaction. With the introduction of potassium hydroxide, some groups were blue-shifted, the -CH_2_-bending vibrational absorption peak in the structure of carboxymethyl cellulose moved to 1448 cm^−1^ and the N-B-stretching vibrational absorption peak of boron nitride moved to 1367 cm^−1^.

### 3.2. Adsorption Mechanism of CMC-BNNS

**Figure 3:**
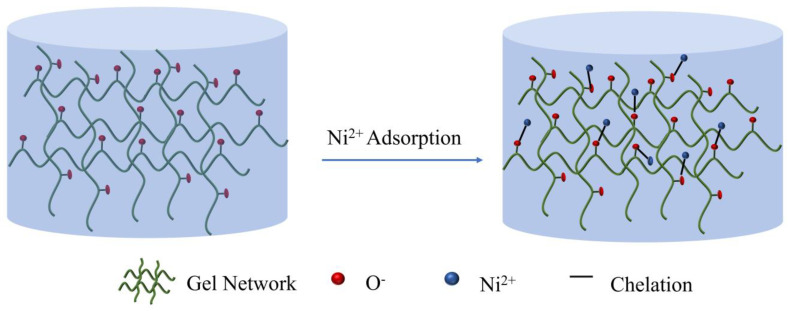
Adsorption and detection mechanisms of CMC-BNNS hydrogel for Ni^2+^.

This experiment was conducted to investigate the effect of the initial concentration of Ni^2+^ on the adsorption capacity of CMC-BNNS. Research on the effect of initial concentration, pH, temperature and adsorption time on the adsorption performance of Ni^2+^ was examined. Figure S1 shows that CMC-BNNS reached the ideal adsorption conditions with a maximum adsorption capacity of 344 mg/g when the initial concentration of Ni^2+^ was 800 mg/g, pH was 4.5, temperature was 25 °C and the adsorption time was 100 min (all adsorption experiments were averaged over three trials and the data were also averaged). Table 1 lists the adsorption effects of four biomass-based adsorbents on nickel ions and compares them with the findings of this article. From the table, it can be seen that CMC-BNNS has a good adsorption effect on Ni^2+^. Langmuir and Freundlich adsorption isothermal models were used to fit the Ni^2+^ adsorption process by CMC-BNNS, and the L model correlation coefficient, R^2^ = 0.9942, was higher than that of the F model correlation coefficient, R^2^ = 0.9232, in both nonlinear and linear fits (Figure 2e). In the linear fit, the L model correlation coefficient was R^2^ = 0.9273, which was higher than the F model correlation coefficient (R_2_ = 0.9265) (Figure S2). Therefore, the L model has a better fitting effect on the adsorption process. This suggests that the adsorption of Ni^2+^ by CMC-BNNS hydrogels mainly relies on monolayer adsorption [34]. The pseudo-first-order and pseudo-second-order adsorption kinetic models were used to fit the Ni^2+^ adsorption data of CMC-BNNS both linearly and nonlinearly. The correlation coefficient of the PSO model, R^2^ = 0.9441, was higher than that of the PFO model correlation coefficient, R^2^ = 0.8990 (Figure 2f). In the linear fit, the PSO model correlation coefficient was R^2^ =0.9932, which is higher than the PFO model correlation coefficient (R^2^ = 0.8018) (Figure S3). According to theoretical calculations, the theoretical adsorption amount of the PSO model was 370.37 mg/g, close to the actual adsorption amount (Table 2). Therefore, the PSO model has a good fitting effect on the adsorption process. This indicates that the adsorption of Ni^2+^ by CMC-BNNS is dominated by chemisorption [35]. The adsorption of Ni^2+^ by CMC-BNNS may be the result of its own good porosity and abundant adsorption sites. CMC-BNNS can be dominated by chemosorption to realize the monomolecular layer adsorption of Ni^2+^ [36]. CMC is the core adsorbent component of the hydrogel, and the carboxylic acid group (-COO-) on its molecular chain dissociates in solution and binds to Ni^2+^ due to Ni^2+^ and the H^+^ in the carboxylic acid group or Na^+^ in a substitution reaction. The oxygen atom of the carboxylic acid group provides the lone pair of electrons needed to form a stable coordination bond with Ni^2+^ (Figure 3). BNNS enhances adsorption properties mainly through its high specific surface area and physical adsorption assistance. The lamellar structure of BNNS enlarges the contact area of the hydrogel to increase the exposure of the active site. Polar B-N bonding on the surface of BNNS may be temporarily trapped by electrostatic gravity Ni^2+^, but the effect is weak and reversible and the main contribution still comes from the chemisorption of CMC.

**Figure 4:**
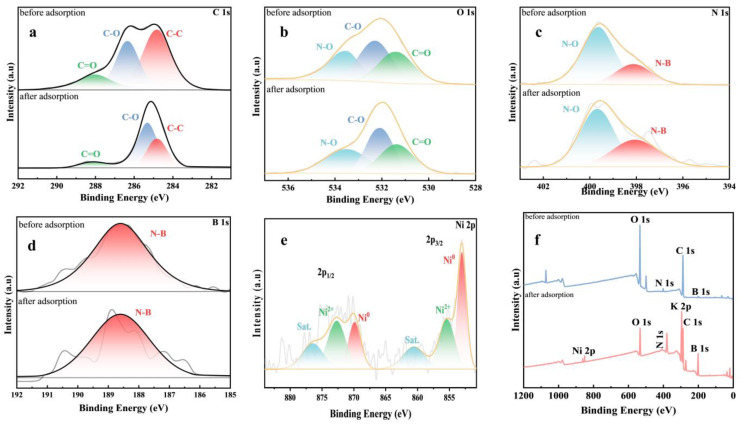
XPS spectra of CMC-BNNS gels before and after Ni^2+^adsorption. (**a**) XPS high-resolution spectrogram after adsorption. C 1s (**b**), O 1s (**c**), N 1s (**d**) and B 1s (**e**) XPS survey spectra of NCDs-SP-MCC-gels after Ni^2+^adsorption. (**f**) XPS survey spectra.

The adsorption of Ni^2+^ by CMC-BNNS was further analyzed with the help of XPS (Figure 4). In the carbon spectrum, the decrease in the C-O bond binding energy when the C-O bond moved from 286.3 ev to 285.4 ev indicated that the bond was involved in the chemical reaction. In addition, the position of the N-O and B-N bond peaks remained unchanged, indicating that these two chemical bonds were not involved in the Ni^2+^ adsorption reaction, and that the N-O and B-N bonds functioned to maintain the stability of the structure of the CMC-BNNS hydrogel, which further suggests that the structure of the carboxymethylcellulose and boron nitride nanosheets will not be damaged with the adsorption of Ni^2+^ [37]. In the full spectrum of the XPS, the O elemental peak moved from 532 ev to 531 ev, the elemental N peak moved from 400 ev to 377 ev, and the elemental C peak moved from 287 ev to 284 ev; the intensity of all peaks decreases after adsorption of Ni^2+^. Ni^2+^ occupied the active sites on the surface of the CMC-BNNS hydrogel material, resulting in a decrease in the photoelectron emission intensity of the original surface elements, and the combination of Ni2p (Figure 4e) further suggests that the Ni^2+^ was successfully adsorbed.

**Table 1:** Comparison of adsorption properties of different materials for Ni^2+^.

Sample	Ni^2+^ (mg/g)	Author
GAMAAX	80.75	[38]
Cell-g-P(AN-co-Sty)	139	[39]
PHG	158.68	[40]
F-IIP	69.93	[41]
Biosorption	3.008	[42]
CMC-BNNS	344	This work

**Table 2:** Adsorption kinetic parameters for CMC-BNNS adsorption of Ni^2+^.

Metals	Parameters	Pseudo−First−Order Model		Pseudo−Second−Order Model	
Ni^2+^	*R* ^2^	0.8018		0.9932	
	Constants	*k*_1_	0.01113 min^−1^	*k*_1_	0.00015 g/(mg·min)
		*q_e_*	181.241 mg/g	*q_e_*	370.37 mg/g

### 3.3. Transformational Utilization of CMC-BNNS Hydrogels

**Figure 5:**
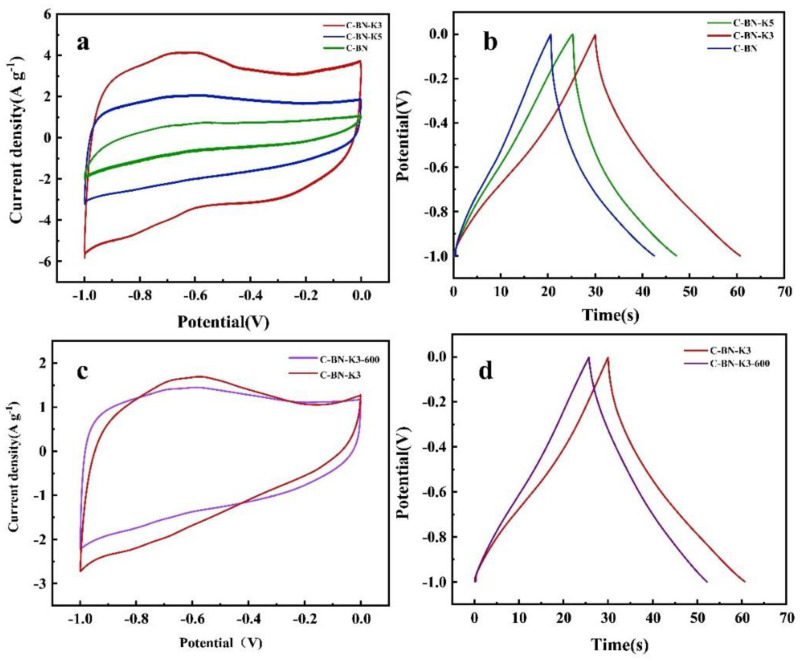
Electrochemical performances of EDLC materials with different Ni^2+^ concentrations and activation temperatures, measured in a three-electrode system with Hg/HgO as the reference electrode. (**a**,**c**) CV curves for EDLC materials at 5 mV/s in the potential window of −1~0 V. (**b**,**d**) GCD curves at a current density of 1 A/g.

In the above study, it can be seen that CMC-BNNNS hydrogels demonstrate a good adsorption of Ni^2+^. However, adsorbent materials containing Ni ions are still hazardous solid waste, and they have the potential to negatively impact the ecosystem. Ni^2+^ is commonly used in supercapacitors, and KOH is a commonly used activation material [43]. Therefore, we impregnated adsorption-saturated CMC-BNNNS hydrogels with different concentrations of KOH and immobilized Ni ions in the carbon material using a two-step activation pyrolysis method. The electrochemical properties of the prepared synthetic materials were systematically evaluated using 6 M KOH aqueous solution in a three-electrode system. The comparative electrochemical results of the synthesized materials are analyzed in Figure 5. For the activated carbon material, the CV curve (Figure 5a) at 50 mV/s exhibited a near-rectangular-type trajectory with a slight deformation. This figure shows typical double electric layer capacitor (EDLC) characteristics. Among the synthesized materials, the C-BN-Ni-K3 curve has the highest area under the curve with a high current response. The curve indicates high energy storage performance and further capacitance enhancement of C-BN-Ni-K3. The GCD curve in Figure 5b exhibits symmetric triangular behavior at 1 A/g current density, where the iR drop is negligible. C-BN-Ni-K3 represents reversible behavior under ideal EDLC electrodes and improved ionic diffusion kinetics at the electrode–electrolyte interface. The GCD curve in Figure 5b shows symmetric triangular behavior with a negligible drop in IR [44].

From the GCD analysis, it can be concluded that the optimal capacitance of C-BN- Ni-K3 is 40.6 F/g, while the capacitances of C-BN-Ni and C-BN-Ni-K5 are 21.8 F/g and 25.3 F/g, respectively. This indicates that in the absence of KOH, the activation effect of C-BN-Ni is not achieved and the pore structure is reduced. In addition, a high KOH concentration erodes the carbon layer, collapsing the C-BN-Ni pore structure and leading to an increase in its own pore size [45]. The effects of different factors on BET are further shown in Table 3. The comparison of C-BN-K3, C-BN-K5 and C-BN shows that the high concentration of KOH eroded the carbon layer, the volume and area of the micropores were greatly reduced, the average pore diameter increased and the specific surface area was reduced; the activation of the pore volume and area without KOH also decreased, the average pore diameter increased and a comparison could be made between C-K3 and C-BN-K3. The addition of boron nitride significantly increased the area of the micropores, which resulted in an increase in specific surface area, and the layered structure of boron nitride can be used as a physical template to guide the material to form a uniform pore distribution. The interlayer gap provides the initial space for the formation of micropores. In addition, the inertness of boron nitride can slow down the drastic etching of KOH to avoid the merging of micropores into medium/large pores and optimize the pore size distribution. In the activation process, the formation of the porous structure is mainly attributed to the formation of K_2_CO_3_ and metal K, which etches the carbon skeleton and generates the pore network structure. K^+^ is embedded between the material layers, leading to structural expansion and subsequent gas release (e.g., H_2_, CO_2_) to further expand the pore network. For the conventional activation process, the tube furnace temperature was increased directly from room temperature to the target activation temperature at a heating rate of 5 °C/min. In contrast, the addition of a pre-activation process at 300 °C provides a longer residence time, allowing for a more complete activation reaction. In the 300 °C pre-activation process, the activation reaction mainly consists of KOH dehydration: 2KOH → K_2_O + H_2_O [46]. After KOH dehydration, the carbon layer can be expanded by the presence of K_2_O.

C + H_2_O → CO + H_2_

CO + H_2_O → CO_2_ + H_2_

CO_2_ + K_2_O → K_2_CO_3_

K_2_CO_3_ + 2C → 2K + 3CO

C + K_2_O → 2K + CO

**Figure 6:**
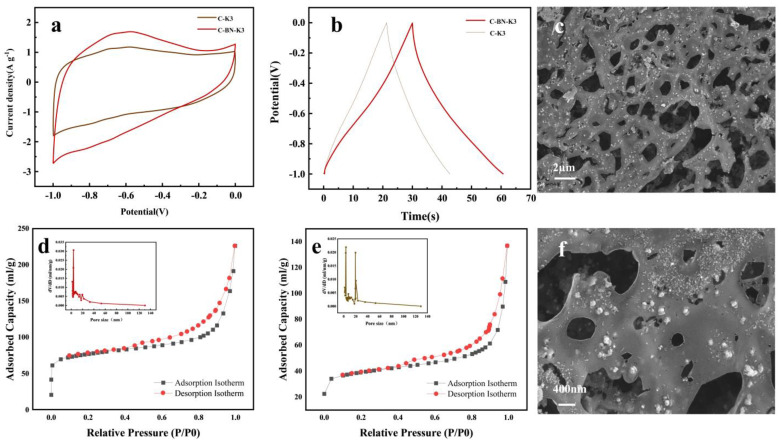
(**a**) CV curves at a scan rate of 50 mV/s; (**b**) GCD curves at 1 A/g; BET test for nitrogen adsorption−desorption isothermals and pore size distribution curves (**d**) C-BN-K3; (**e**) C-BN; (**c**,**f**) SEM images of C-BN-K3.

During the activation process at 600 °C, K_2_O is converted to metal and produces a pore network structure dominated by micropores. The pore structure of the C-BN- Ni material can be further observed by SEM in Figure 6c. There are many pores in the structure of the C-BN-Ni material, and a large number of fine nanocrystalline nickel particles are dispersed on the surface and within the pores of the C-BN-Ni material. Figure 6f shows a large number of nanoparticles aggregated at the porous channels, a phenomenon that can further assist in the formation of the pore structure of the material [47]. In addition, most of the nickel particles immobilized on the surface of the carbon material show a tendency to fuse with the carbon material.

### 3.4. Study of the Electrical Properties of C-BN-K3

**Figure 7:**
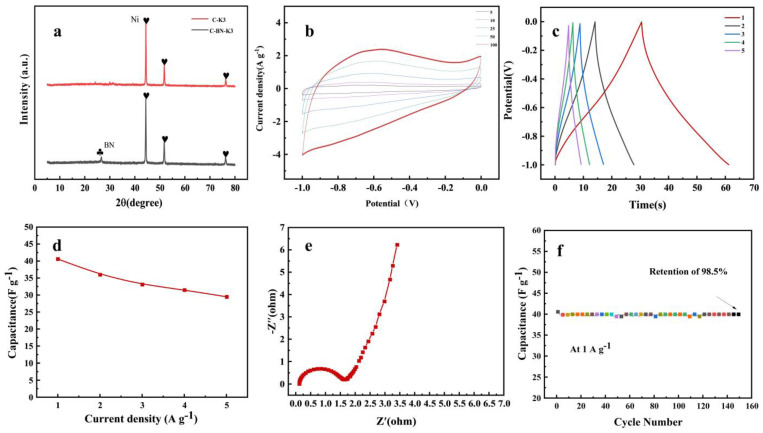
(**a**) The XRD patterns of materials; (**b**) CV curves at different scan rates from 5 to 100 mV/s; (**c**) GCD curves under different current densities from 1 to 5 A/g; (**d**) specific capacitances at different charge−discharge current densities; (**e**) nyquist plots in 6 M KOH over the frequency range of 100 kHz to 1 Hz; (**f**) cycling stability at the current density of 1 A/g.

Based on the above results, C-BN-K3, with the best capacitive properties, was selected for electrochemical characterization. XRD showed Ni and BN characteristic peaks (Figure 7a), verifying that the nickel particles were successfully immobilized in the carbon material structure. All nickel oxides were reduced during calcination in a tube furnace with the following reaction equation:

Ni(OH)_2_ → NiO + H_2_O

NiO + C → Ni + CO

NiO + CO → Ni + CO_2_

In the bet analysis, it can be seen that the unadded boron nitride nearly doubles the surface area of the C-BN-K3 material (Table 3). The addition of boron nitride material increases the microporous structure of C-BN-K3. Figure 6d,e and Figure S4 show that the prepared carbon materials all belong to H4-type hysteresis rings. In addition, the carbon material mainly consists of micropores and mesopores, where the micropores accumulate charge for the formation of double-layer capacitor structures and the presence of mesopores facilitates charge transport [46].

Good reversibility of the electrode material was observed from the CV curves of C-BN-K3. The density of the electrode material at different scan rates from 5 mV/s to 100 mV/s indicates that the material can be used properly in high current density conditions (Figure 7b). The electrode cyclic voltammetry curve also shows a similar rectangular shape at higher scan rates, which is an electrode characteristic of a double electric layer capacitor. This electrode feature reveals that the electrolyte penetrates easily into the active sites inside the electrode material and forms an effective double electric layer. It also indicates the low-resistance and high-power capacitance characteristics of the electrode material. When comparing the GCD curves at different current densities of 1 A/g, 2 A/g, 3 A/g, 4 A/g and 5 A/g (Figure 7c) and calculating the specific capacitance at different current densities, the specific capacities are similar; the above indicates the feasibility of preparing electrode materials with a nickel-containing waste adsorbent (Figure 7d) [48]. In addition, electrochemical impedance spectroscopy was performed to study the C-BN-K3 electrode’s electrochemical performance. As shown in Figure 7e, the capacitance of the C-BN-K3 electrode was still 98.6% of the original capacitance after 150 constant voltage cycles (Figure 7f).

## 4. Conclusions

CMC-BNNS hydrogel has good adsorption performance for nickel ions. When the initial concentration of Ni^2+^ was 800 mg/g, the pH of the aqueous solution was 4.5, the temperature was 25 °C and the adsorption time was 100 min, the material reached the ideal adsorption state, and the maximum adsorption amount was up to 344 mg/g. The adsorption process of nickel ions on the CMC-BNNS hydrogel conformed to the Langmuir isothermal model and the pseudo-second-order kinetic model. After adsorption, the waste gel was converted into nickel-doped porous carbon material through carbonization, and this was used as the electrode of a double-layer capacitor. In the electrode performance study, KOH activation significantly affected the energy storage characteristics of the C-BN-Ni electrode and the boron nitride material enhanced the capacitance performance. The specific capacitance of the C-BN-Ni-K3 electrode reached 40.6 F/g at 1 A/g current density, and the capacity remained stable after 150 cycles. This study creatively and simultaneously solves the problems of nickel-containing wastewater purification and environmental pollution caused by waste adsorbents, and confirms the application value of the “adsorption-resourcefulness” of nickel as a heavy metal pollutant.

## Figures and Tables

**Table 3:** Summary of the BET parameters for the prepared samples.

	BET Surface Area (m^2^/g)	Micropore Volume (cm^2^/g)	Micropore Area (m^2^/g)	Average Pore Size (nm)
C-K3	125.5 ± 2.5	85.4 ± 4.3	0.046 ± 0.002	6.53 ± 0.33
C-BN-K3	241.8 ± 4.8	187.5 ± 9.4	0.098 ± 0.005	5.03 ± 0.25
C-BN-K5	96.4 ± 1.9	54.8 ± 2.7	0.029 ± 0.001	9.21 ± 0.46
C-BN	215.7 ± 4.3	179.2 ± 9.0	0.096 ± 0.005	6.28 ± 0.31
C-BN-K3-600	238.3 ± 4.8	182.2 ± 9.1	0.094 ± 0.005	5.04 ± 0.25

Note: Error ranges for BET surface area are based on an instrument precision of ±2%. Error ranges for micropore volume, micropore area and average pore size are estimated based on the literature data and instrument performance, and are typically within ±5%.

## Data Availability

Data are contained within the article and supplementary materials.

## Supplementary Materials

The following supporting information can be downloaded at: https://www.mdpi.com/article/10.3390/polym17050567/s1, Figure S1. Effect of (a) the initial Ni^2+^ (b) adsorption temperature. (c) pH and (d) adsorption time; Figure S2. Langmuir (a) Freundlich (b) linear fitting; Figure S3. Pseudo-second order (a) Pseudo-first order (b) linear fitting; Figure S4. BET test for Nitrogen adsorption–desorption isothermals and pore size distribution curves (d) C-BN (e) C-BN-K3-600; Figure S5. The XRD patterns of materials; Table S1. Adsorption isotherms parameters for CMC-BNNS adsorption Ni^2+^. Reference [26] is cited in Supplementary Materials.
