# Supplementary material for: Adsorption of Aqueous Nickel Ion by Biomass Carboxymethyl Cellulose-Doped Boron Nitride Composites and Its Subsequent Energy Storage
Source: Polymers (Basel). 2025 Feb 20;17(5):567. doi: 10.3390/polym17050567 (PMC11902729; doi:10.3390/polym17050567)
Supplement: Supplementary file 1 [file polymers-17-00567-s001.zip › polymers-3448706-supplementary.pdf]

Supporting Information

Adsorption of Aqueous Nickel Ion by Biomass Carboxymethyl Cellulose-Doped Boron Nitride Composites and Its Subsequent Energy Storage

Xinran Li [1], Boyun Wang [1], Wanqi Zhang [2], Xiaotao Zhang [1,3,4,*] and Ximing Wang [2,3,*]

[1] College of Science, Inner Mongolia Agricultural University, Hohhot 010018, China
[2] College of Material Science and Art Design, Inner Mongolia Agricultural University, Hohhot 010018, China
[3] Inner Mongolia Key Laboratory of Sandy Shrubs Fibrosis and Energy Development and Utilization, Hohhot 010018, China
[4] National Forestry Grassland Engineering Technology Research Center for Efficient Development and Utilization of Sandy Shrub, Hohhot 010018, China
* Correspondence: lianzixiaotao@163.com (X.Z.); wangximing@imau.edu.cn (X.W.)

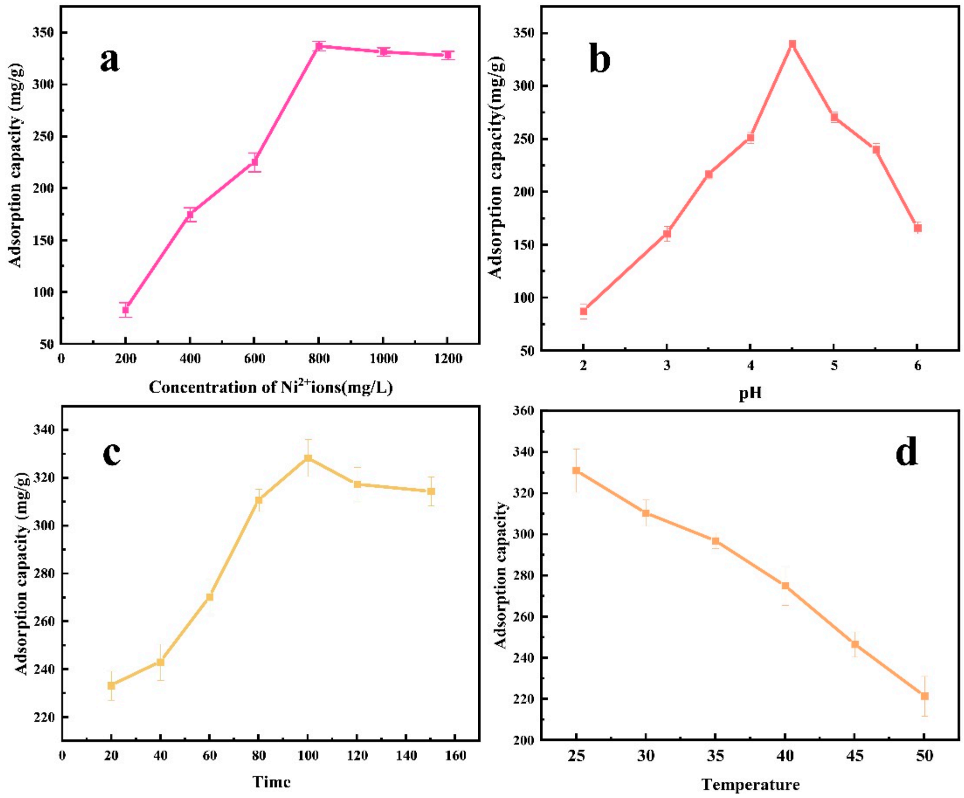

Fig. S1 Effect of (a) the initial Ni^{2+}(b) adsorption temperature (c) pH and (d) adsorption time

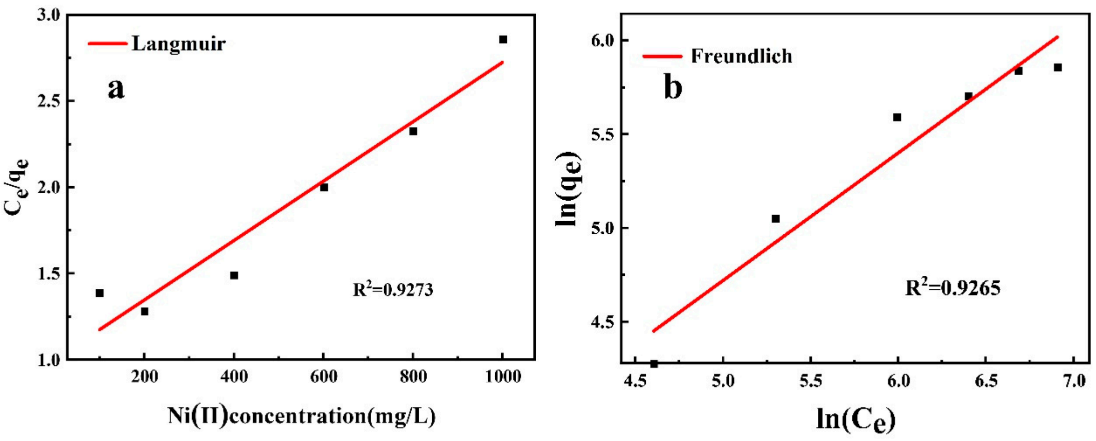

Fig. S2 Langmuir(a) Freundlich(b) linear fitting

Adsorption isotherm model equation as following shows[1]。

Langmuir model:

$$qe = \frac{K_L q_e C_e}{1 + K_L C_e}$$

Freundlich model:

$$qe = K_F C_e^{\frac{1}{n}}$$

where q_e (mg/g) is the monolayer saturation adsorption capacity; C_e (mg/L) is the concentration of metal ions at equilibrium; K_L (L/mg) is the Langmuir constant the Freundlich constant, 1/n is the value used to indicate the heterogeneity of the interface; q_e (mg/g) is the adsorption capacity at equilibrium.

Table. S1 Adsorption isotherms parameters for CMC-BNNS adsorption Ni^{2+}

Metals	Parameters	Langmuir model		Freundlich model	
	R^2	0.9273		0.9265	
Ni^{2+}	Constants	K_L	0.0017 L/mg	K_F	3.714 L/g
		q_e	581.39 mg/g	1/n	0.6812

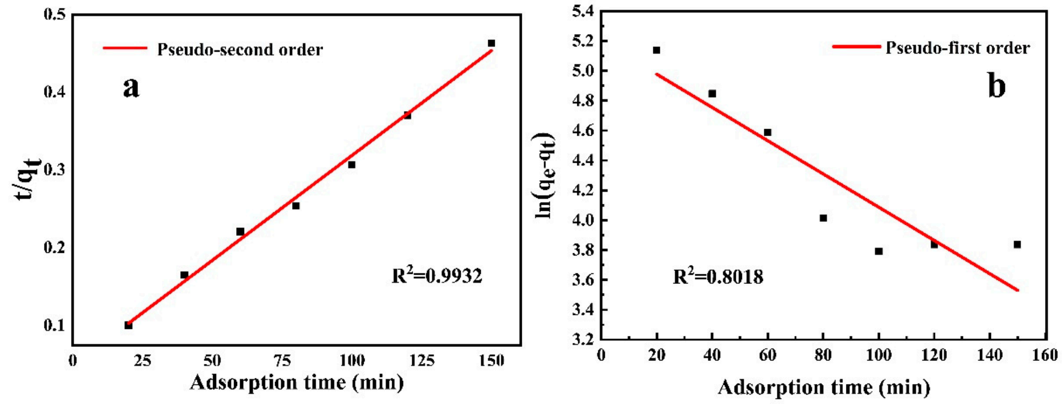

Fig. S3 Pseudo-second order(a) Pseudo-first order (b) linear fitting

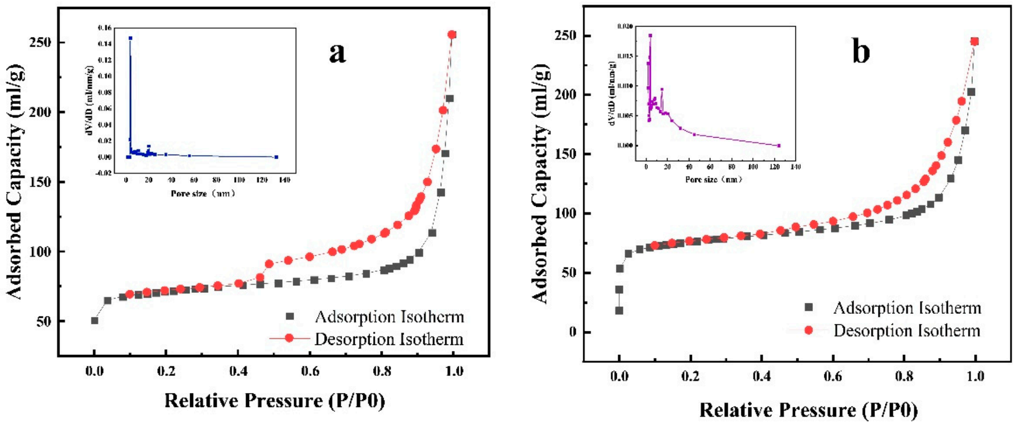

Fig. S4 BET test for Nitrogen adsorption–desorption isothermals and pore size distribution curves (d)C-BN (e) C-BN-K3-600

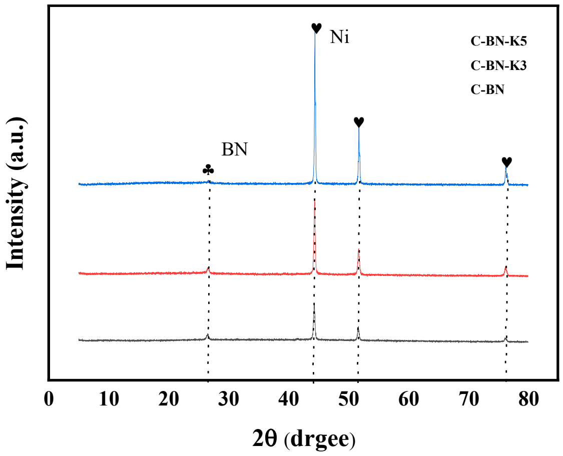

Fig. S5 The XRD patterns of materials

References:

1. Wang, J.; Wei, X.; Kong, H.; Zheng, X.; Guo, H., Hydrothermal Ammonia Carbonization of Rice Straw for Hydrochar to Separate Cd(II) and Zn(II) Ions from Aqueous Solution. *Polymers* **2023,** *15* (23).
